# Chronic infections can generate SARS-CoV-2-like bursts of viral evolution without epistasis

**DOI:** 10.1093/ve/veag008

**Published:** 2026-02-18

**Authors:** Edwin Rodríguez-Horta, John Strahan, Aaron R Dinner, John P Barton

**Affiliations:** Department of Computational and Systems Biology, University of Pittsburgh School of Medicine, 4200 Fifth Ave, Pittsburgh, PA 15260, United States; Department of Chemistry and James Franck Institute, University of Chicago, 929 E 57th Street, Chicago, IL 60637, United States; Department of Chemistry and James Franck Institute, University of Chicago, 929 E 57th Street, Chicago, IL 60637, United States; Department of Computational and Systems Biology, University of Pittsburgh School of Medicine, 4200 Fifth Ave, Pittsburgh, PA 15260, United States

**Keywords:** SARS-CoV-2, variants of concern, evolution, mathematical modeling, epidemiology

## Abstract

Multiple SARS-CoV-2 variants have arisen during the first years of the pandemic, often bearing many new mutations. Several explanations have been offered for the surprisingly sudden emergence of multiple mutations that enhance viral fitness, including cryptic transmission, spillover from animal reservoirs, epistasis between mutations, and chronic infections. Here, we simulated pathogen evolution combining within-host replication and between-host transmission. We found that, under certain conditions, chronic infections can lead to SARS-CoV-2-like bursts of mutations even without epistasis. Chronic infections can also increase the global evolutionary rate of a pathogen even in the absence of clear mutational bursts. Overall, our study supports chronic infections as a plausible origin for highly mutated SARS-CoV-2 variants. More generally, we also describe how chronic infections can influence pathogen evolution under different scenarios.

## Introduction

During the SARS-CoV-2 pandemic, multiple variants of concern (VOC) have arisen and spread widely throughout the human population, driving waves of infections and mortality (Harvey et al. 2021, Mascola et al. 2021, Peacock et al. 2021, Tabatabai et al. 2023). The spread of new VOCs has been facilitated by their ability to evade adaptive immunity developed by previous infections or vaccines (Salehi-Vaziri et al. 2022, Carabelli et al. 2023). VOC mutations can also increase virus transmissibility in other ways, such as by improving the receptor binding ability of the viral Spike protein or increasing viral load (Salehi-Vaziri et al. 2022, Carabelli et al. 2023).

A singular and unexpected characteristic of early VOCs has been their abrupt emergence. New variants such as Alpha, Delta, and Omicron appeared bearing many mutations that had not been previously observed, seemingly making a large evolutionary leap compared with co-circulating variants. This phenomenon is surprising given the tight transmission bottlenecks inferred for SARS-CoV-2 (Braun et al. 2021, Bendall et al. 2023). During acute infections, few mutations are produced and even fewer are expected to be passed on in new infections (Braun et al. 2021, Bendall et al. 2023). In principle, one would then expect viral evolution to proceed through the gradual accumulation of advantageous (i.e. transmission-increasing) mutations.

Multiple hypotheses have been put forward to explain the sudden appearance of a new, highly transmissible variant with a large number of novel mutations (Markov et al. 2023). One possibility is cryptic transmission, where undetected circulation in humans allows for long-term viral evolution (Adepoju 2021, Davis et al. 2021, Wilkinson et al. 2021). However, given the number of novel mutations observed in VOCs, this scenario would require that variants remain undetected for long periods of time. Circulation in animal reservoirs, followed by subsequent spillover to humans, could also explain the sudden appearance of VOCs with many mutations (Bashor et al. 2021, Lu et al. 2021, Hale et al. 2022, Pickering et al. 2022). As an alternative to hidden circulation in unobserved human or animal populations, epistasis (i.e. non-additive effects of mutations on viral transmissibility) has been cited as a possible factor underlying VOC emergence (Zahradník et al. 2021, Ghafari et al. 2022, Martin et al. 2022, Smith and Ashby 2022). If multiple mutations are needed to confer a significant fitness advantage to the virus, then variants with a small number of mutations may not be observed at high frequencies in humans.

Based on clinical data, chronic SARS-CoV-2 infections have emerged as a plausible source of highly divergent variants. Typically, acute SARS-CoV-2 infections resolve within days to weeks. However, in some individuals, chronic infections can persist for months. Chronically infected individuals often have compromised immune systems that are unable to fully clear infections (Avanzato et al. 2020, Choi et al. 2020, Clark et al. 2021, Lee et al. 2022). During chronic infections, there is sufficient time for SARS-CoV-2 to generate multiple mutations, which can rise in frequency and ultimately fix in the viral population within that individual. Genomic analyses have shown that the rate of accumulation of mutations within chronically infected individuals is higher than the rate of SARS-CoV-2 evolution between individuals (Chaguza et al. 2023). In addition, VOC mutations have been observed in chronically infected individuals (Wilkinson et al. 2022, Chaguza et al. 2023). Accelerated selection of antibody evasion mutations has also been observed in long-term infections treated with monoclonal antibodies or convalescent plasma (Kemp et al. 2021).

Given the potential importance of chronic infections in the evolution of SARS-CoV-2, mathematical modellers have begun exploring its anticipated epidemiological effects in theory and simulations (Van Egeren et al. 2021, Ghafari et al. 2022, Kumata and Sasaki 2022, Smith and Ashby 2022). Recent work has incorporated immunocompromised hosts into susceptible/infected/recovered epidemiological models that also include some component of within-host viral evolution (Kumata and Sasaki 2022, Smith and Ashby 2022). Smith and Ashby predicted that large jumps in the proportion of novel variants should only be observed when there is a significant amount of epistasis between immune escape mutations and a sufficient proportion of the population is immunocompromised. In other words, the role of immunocompromised hosts in this model is to allow the virus to cross epistatic fitness valleys. Additional work has also considered fitness valley crossing for infections of different durations, but without modelling effects on transmission (Van Egeren et al. 2021).

In an extensive study, Ghafari et al. (2022) considered the effects of chronic infection on the emergence of highly transmissible VOCs. In their model, VOC mutations fix at a constant rate within chronically infected hosts. They consider multiple fitness landscapes for transmission between individuals, including models where VOC mutations make equal, additive contributions to transmission and ‘plateau-crossing’ models where mutations have small effects until a critical number are accumulated. They concluded that chronic infection could facilitate the emergence of VOCs, defined as variants with specific transmission-increasing mutations, especially with plateau-crossing fitness landscapes.

Here, we develop a generic model of pathogen evolution, coupling evolution within hosts and transmission between individuals. The primary goal of our model is to understand how chronic infections can affect pathogen evolutionary dynamics over long times. Using transmission effects of mutations inferred from SARS-CoV-2 data (Lee et al. 2025), we show that bursts of mutations like those observed during the pandemic can occur even with a simple, additive fitness landscape. In particular, we explore how the within-host mutation rate, typical duration of infection, and fraction of infections that are chronic affect the likelihood of mutational bursts. We find that bursty evolution is especially likely when the acute infection time is short compared with the duration of chronic infections. Our results highlight scenarios in which chronic infections produce evolutionary dynamics that are qualitatively different from those that are observed in most simple evolutionary models.

## Materials and methods

### Model of pathogen evolution within and between hosts

**Figure 1:**
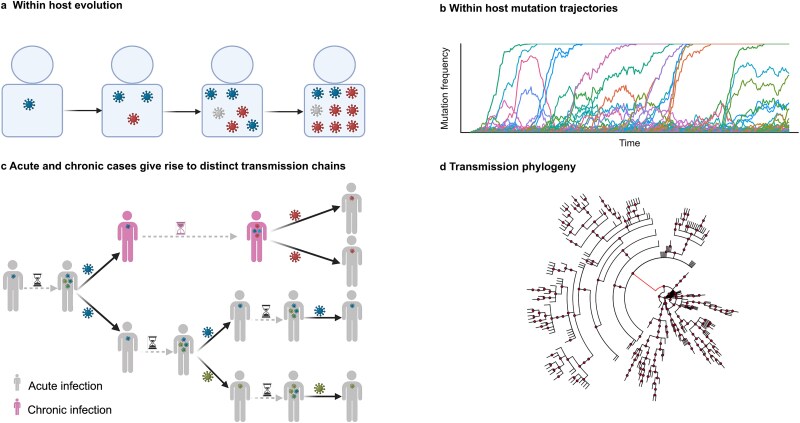
Global evolutionary model for intra-host and between-host levels of evolution. (a) The viral population, begins with a single starting genotype and undergoes discrete and non-overlapping generations of Wright–Fisher evolution subject to mutation, selection, and genetic drift. (b) Schematic frequency of viral mutations over time within an individual host. (c) The population of individuals comprises patients with acute infection and patients with chronic infections. Viral diversity is generated during intrahost evolution. During transmission between acutely infected hosts, most mutations are lost due to tight transmission bottlenecks. Chronic infection can allow for the evolution and transmission of a highly divergent variant. (d) Example phylogeny of between-host transmission, with a long red branch indicating transmission from a chronically infected individual. The phylogeny is built from donor-infected transmission pairs, with branch lengths representing generation times. Figures (a) and (c) were created in BioRender.com.

The global evolutionary dynamics of pathogens such as SARS-CoV-2 are a consequence of processes that occur within and between infected individuals. Evolution within individuals generates a genetically diverse cloud or ‘quasispecies’ of variants (Eigen et al. 1988, Coffin 1995, Domingo et al. 2012). Differential transmission of variants between hosts ultimately results in pathogen evolution across individuals. We include both levels of evolution in our model (Fig. 1).

To model the emergence and accumulation of mutations within each host, we use a standard, stochastic Wright–Fisher model (Ewens 2012). We assume that the pathogen population begins with a single starting genotype—consistent with tight transmission bottlenecks—and evolves in discrete generations subject to selection, mutation, and genetic drift. In each replication cycle, neutral and positively selected mutations are randomly introduced with rates ${\mu}_N$ and ${\mu}_B$, respectively. These mutation rates represent combinations of the basic probability per replication cycle that a new mutation is introduced and the probability that the mutation is beneficial or neutral. We assume that significantly deleterious mutations are rare enough to be efficiently eliminated by selection, and do not model them explicitly.

The distribution of fitness effects of beneficial and neutral mutations was derived from selection coefficients learned from SARS-CoV-2 temporal genomic data (Lee et al. 2025) (see Supplementary Fig. S1). In our model, the fitness effects of mutations are additive, so that the net increase or decrease in fitness ${s}_a$ for a virus $a$ with $n$ mutations is


1 \begin{eqnarray*} {s}_a=\sum_{i=1}^n{s}_i. \end{eqnarray*}

Here, the ${s}_i$ are the selection coefficients that quantify the fitness effect of each mutation $i$. A positive selection coefficient indicates a beneficial mutation that increases fitness, while a negative coefficient indicates a deleterious mutation.

We assume that mutations that improve viral replication within a host also improve transmission between individuals. In principle, the effects of mutations on replicative fitness and transmission fitness can be different (Wargo and Kurath 2012). As an example, some immune escape mutations generated during HIV-1 infection can be deleterious in other contexts, causing them to revert when the virus is transmitted to a new host (Allen et al. 2004, Friedrich et al. 2004, Leslie et al. 2004, Zanini et al. 2015, Gao and Barton 2025). However, within-host mutations that produce fitness gains for replication and increase viral load can contribute to increased transmission, as has been shown for Spike mutations in SARS-CoV-2 (Korber et al. 2020, Yurkovetskiy et al. 2020, Liu et al. 2022). Furthermore, VOC mutations have been observed within individuals, including adaptive mutations concentrated in the Spike protein’s receptor binding domain and N-terminal domains (Wilkinson et al. 2022, Chaguza et al. 2023). We have therefore aligned selection pressures within and between hosts for simplicity. Even in this simple case, complex evolutionary dynamics can occur.

We model transmission between individual donors and receptors of infection using a branching process that considers superspreading. In our model, the number of secondary infections is drawn from a negative binomial distribution ${P}_{\mathrm{NB}}\left(k,k/\left(k+{R}^i\right)\right)$, with $k$ the dispersion parameter and ${R}^i=\overline{R}\left(1+{\left\langle s\right\rangle}^i\right)$ the effective reproductive number associated with donor $i$. The negative binomial distribution has been used to model superspreading in past studies of viruses, such as SARS and SARS-CoV-2 (Irwin 1954, Griffiths 1973, Lipsitch et al. 2003, Lloyd-Smith et al. 2005, Althouse et al. 2020). Here, $\overline{R}$ and ${\left\langle s\right\rangle}^i$ are the average baseline (reference) reproductive number and average fitness of the virus population from the donor host, respectively. In our model, we assume that there is always an abundant supply of susceptible hosts, and thus we only explicitly track currently infected individuals. After infection is transmitted, donors are assumed to be recovered and are removed from the infected population. New infections are established by a single pathogen sampled from the donor quasispecies distribution. Thus, most of the variant diversity previously generated is lost. This mimics the characteristic narrow transmission bottleneck observed in some pathogens, including SARS-CoV-2 (Braun et al. 2021, Bendall et al. 2023).

The time between when an individual is first infected and when the infection is transmitted to a new host, which we refer to as the generation time, constrains the level of viral genetic diversity that can accumulate and be transmitted. The generation time varies based on the nature of the infection. Most infections are acute and cleared by the immune system in a short period, parameterized by ${t}_a$. In contrast, infections in immunocompromised hosts can persist far longer, becoming chronic. We define the extended generation time for these chronic infections as ${t}_c$, with ${t}_c\gg{t}_a$. For SARS-CoV-2, we assume an effective replication rate of two cycles per day throughout the course of infection, whether acute or chronic. This choice is supported by experimental measurements showing that a full replication cycle, from cell entry to release of new infectious virions, takes ~10 h (Bar-On et al. 2020), consistent with an average of about two cycles per day. A longer generation time allows for more rounds of viral replication, facilitating the accumulation of genetic diversity. Each time an infection is transmitted, we take the probability that the new host develops a chronic infection to be ${p}_c\ll 1$. The probability that a new infection is of short duration (i.e. acute) is then $1-{p}_c$.

### Simulating pathogen evolution

We simulated multiple realizations of the evolutionary model over 1000 days. At each simulation time, we recorded the average number of mutations in transmitted variants (variants randomly sampled from within-host populations that are transmitted in new infections) and the number of chronically infected individuals across individual populations. Generation times for acute cases were set between 2 and 9 days, covering the values estimated from known infector–infectee transmission pair data or household data across different continents (Chen et al. 2022, Hart et al. 2022). For chronic cases, where generation times are less well determined, we sampled them from a log-normal distribution with mean ${\mu}_L=150$ days and SD ${\sigma}_L=80$ days. We maintained a fixed neutral mutation supply rate of ${\mu}_N={10}^{-4}$ mutations/cycle, based on the underlying mutation rate of SARS-CoV-2 (Bar-On et al. 2020) times the fraction of nonsynonymous mutations found to be neutral in the selection coefficient estimate from SARS-CoV-2 time-series data (Lee et al. 2025) (see Supplementary Information). We varied the beneficial mutation supply rate to explore its effect on pathogen evolutionary dynamics.

## Results

### Patterns of mutation accumulation

**Figure 2:**
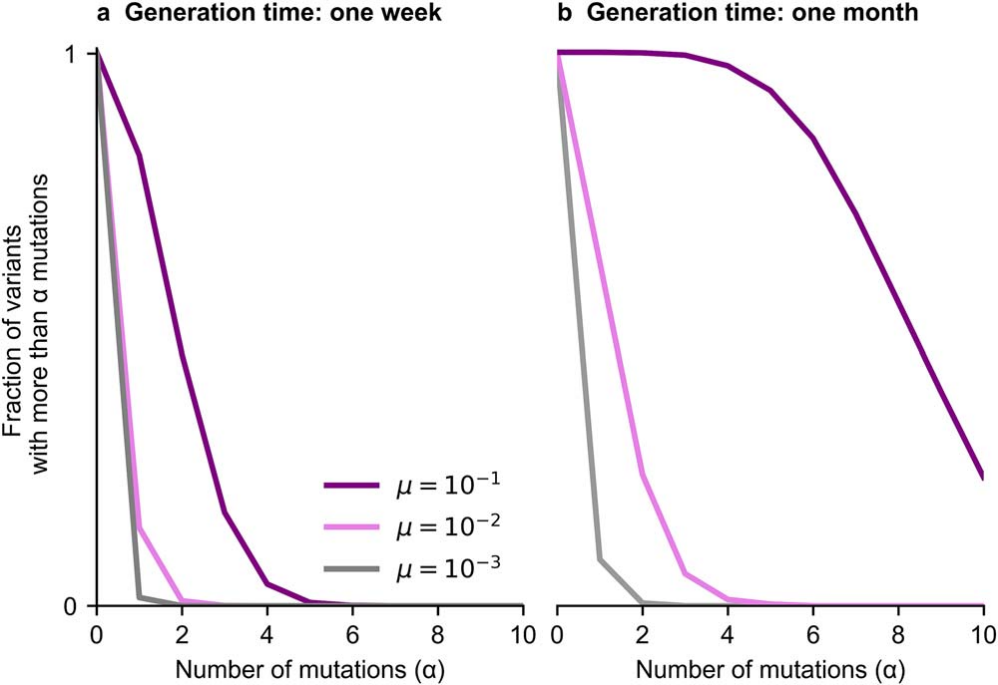
Genetic diversity after typical generation times of acute and chronic infections. (a), Fraction of variants in the intra-host viral population that acquires more than $\alpha$ mutations over one week of infection. (b) Distribution of accumulated mutations after one month of infection. Higher mutation rates lead to the accumulation of more mutations. We consider the same rate, $\mu$, for beneficial or neutral mutations.

Within infected individuals, mutations accumulate progressively in viral populations over time (Fig. 2). Higher mutation rates naturally lead to a more rapid accumulation of mutations. Longer generation times (i.e. more generations of within-host evolution) also allow for more genetic diversity to accumulate within individuals, which can then potentially be transmitted to new hosts.

**Figure 3:**
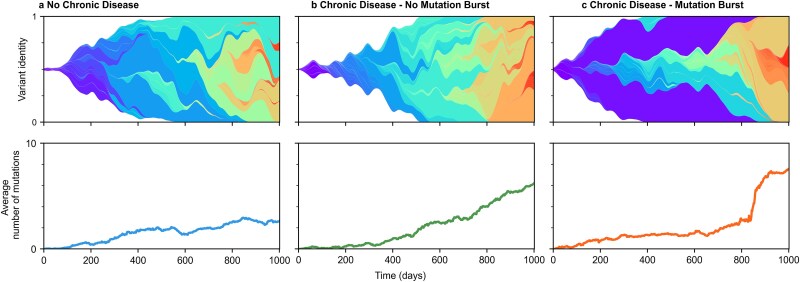
Evolution of viral variants under different scenarios. (a) Dynamic evolution of variants within a population exclusively composed of acute cases. (b) Population consisting of both acute and chronic cases but without mutation burst. (c) Population consisting of both acute and chronic cases with one mutation burst at *t* = 850 days. **Top panels**: Muller plots showing the dynamic evolution of viral variants over time. Each colour represents a distinct variant, with the width of the region proportional to the fraction of individuals harbouring that variant. **Bottom panels**: Corresponding curves showing the average number of accumulated mutations in the population over the same period. For all different simulations, we consider beneficial and neutral mutation rates ${\mu}_B={10}^{-3}$ mutations/cycle and ${\mu}_N={10}^{-4}$ mutations/cycle, respectively. For (b) and (c), chronic cases are included in the percentage per transmission event ${p}_c={10}^{-4}$, and generation times ${t}_c$ are drawn from a log-normal distribution with mean ${\mu}_L=150$ days and SD${\sigma}_L=80$ days. For all cases, the generation time for acute cases is fixed at ${t}_a=4$ days.

Across infected individuals, we found that the evolution of viral populations fell into roughly three patterns (Fig. 3). In cases where there are few or no chronic infections, we observe few viral mutations (Fig. 3a). A few viral lineages tend to dominate the viral population with relatively little competition between them.

When a significant number of chronic infections occur, two distinct outcomes are possible. In one case, the accumulation of mutations in viral populations accelerates, and there is significant competition between viral lineages, but the increase in mutations over time remains roughly linear (Fig. 3b). In other simulations, we observe sudden ‘bursts’ of mutations in viral populations, reminiscent of the emergence of SARS-CoV-2 VOCs (Fig. 3c). These bursts arise when a variant originating in a chronic infection takes over the population, as shown in Supplementary Fig. S2. Burst timing varies substantially among simulations; even under identical parameters, bursts do not occur at consistent times across runs, highlighting the stochastic nature of these events (see Supplementary Fig. S3). Moreover, Supplementary Fig. S4 illustrates that the major contribution to the fitness of such variants often stems from a few key mutations.

The patterns shown in (Fig. 3) correspond to a single simulation replicate for each scenario, yet the same qualitative behaviours are consistently observed across independent simulations. Supplementary Fig. S5 summarizes 1000 replicate runs for each scenario, showing both the mean behaviour and the standard deviation across trajectories. For burst-like events, trajectories are aligned by each replicate’s burst time so that differences in burst magnitude remain visible.

### Parameter landscape for mutational bursts

**Figure 4:**
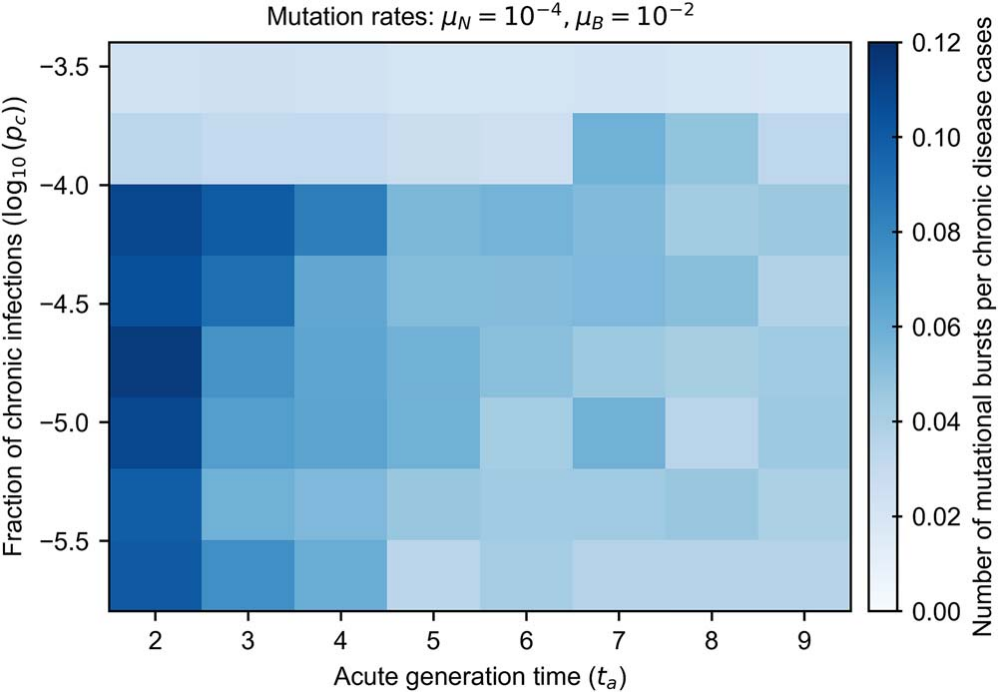
Number of mutational bursts per chronic disease case for beneficial mutation rate of ${10}^{-2}$ mutations/cycle. The frequency of mutational bursts decreases as acute generation times increase because chronic and acute subpopulations exhibit greater similarity, leading to homogenization within individual populations and reducing the likelihood of abrupt mutational events. The dependence on the fraction of chronic infections is nonlinear: While moderate levels of chronic infections lead to more frequent bursts, very high levels cause competition among multiple pathogen variants, each with numerous mutations, reducing the occurrence of isolated bursts. Each value represents an average over 5000 simulations.

To explore how the emergence of mutational bursts depends on model parameters, we mapped the number of mutational bursts per chronic infection across a range of parameter values (Fig. 4). We define a burst as a transient, statistically significant increase in the instantaneous rate of mutation accumulation at the viral population level. To establish a null expectation, we first computed the distribution of maximum mutation accumulation rates across individuals in simulations without chronic infections. Bursts were then identified as intervals in which the rate exceeded this baseline by at least 3.5SD, with the midpoint of each interval taken as the estimated burst time (see Supplementary Information). This approach captures localized accelerations in sequence divergence, analogous to sharp rate increases detected through root-to-tip residuals (Surya et al. 2023) or skyline estimates in phylodynamic analyses (Drummond et al. 2005).

We investigated a wide range of parameters, varying the fraction of chronic infections ${p}_c$, acute generation times ${t}_a$, and rate of beneficial mutations ${\mu}_B$ (see Supplementary Information). For each choice of parameters, we computed the number of mutational bursts per chronic disease case over $5000$ simulations. These results are shown in Fig. 4 for simulations with individual population sizes of around 300 infected individuals at each point in time (see Supplementary Information). Repetition of this analysis with larger populations ($\sim{10}^4$ infected individuals) yields qualitatively similar patterns (Supplementary Fig. S8a).

We found several factors that facilitated the emergence of mutational bursts. Intuitively, bursts occurred more frequently when beneficial mutation rates were higher (see analogous heatmap in Supplementary Fig. S9 for a lower mutation rate). We also found that bursts occurred more frequently when the acute generation time ${t}_a$ was shorter. Longer acute generation times lead to greater similarity in the viral populations in acute and chronically infected individuals, homogenizing the accumulation of mutations and decreasing the likelihood of abrupt increases in mutations.

Interestingly, we found that the likelihood of mutational bursts depends nonlinearly on the fraction of chronic infections. As the fraction of chronic infections increases, new adaptive mutations are generated more frequently and spread throughout the population, making isolated bursts unlikely. At very high frequencies of chronic infections, several pathogen variants with many mutations can be produced simultaneously. These variants then compete among hosts, reducing several potential bursts to a single one (see Supplementary Fig. S10).

### Effects of chronic infections on evolutionary rate

A recent study found that the evolutionary rate of SARS-CoV-2 within a chronically infected individual was higher than the estimated global evolutionary rate of the virus, measured by the rate of substitutions over time (Chaguza et al. 2023). One plausible explanation for this difference is that within-host evolution does not face the same stringent transmission bottlenecks that can slow between-host evolution. In our simulations, we observed that mutational bursts can occur due to the spread of new pathogen variants that evolved for long times within chronically infected individuals. Do chronic infections affect the overall evolutionary rate even in the absence of bursts?

**Figure 5:**
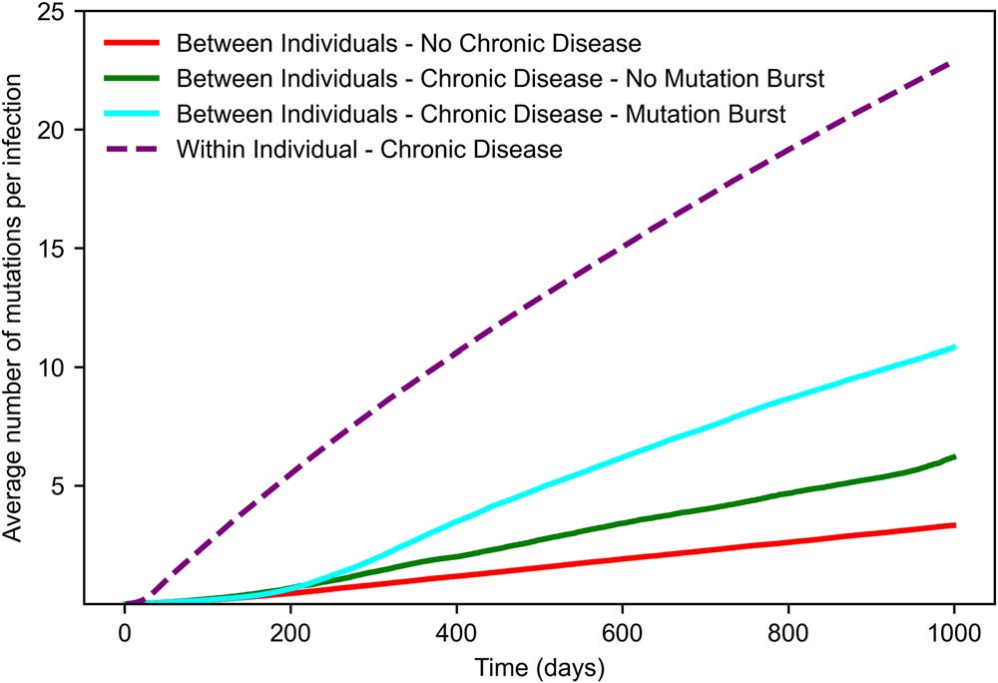
Average number of accumulated mutations per infection in simulations of within-host evolution and between-host evolution with and without chronic infections. For each observation time, the reported value represents the average number of mutations within intra-host viral populations of actively infected individuals, normalized by the total number of infected individuals at that time. The higher evolutionary rate is obtained within a chronically infected individual due to the absence of stringent bottlenecks imposed by transmission events. Between-host evolution with chronic infections, even in the absence of a burst, leads to an increased rate of mutation accumulation compared to populations with only acute infections. The simulations were conducted with a beneficial mutation rate of ${\mu}_B={10}^{-3}$ mutations/cycle, an acute generation time of ${t}_a=2$ days, and a probability of new chronic infection of ${p}_c=4\times{10}^{-4}$. Each curve represents an average of over 5000 simulations.

To answer this question, we quantified, for different scenarios, the rate of mutation accumulation across individuals, in units of substitutions per genome per day, defined as the slope of the average number of accumulated mutations per infection over time (Fig. 5). Specifically, we measured the evolutionary rate within chronically infected individuals and the evolutionary rate between individuals in three different cases: in simulations with no chronic infections (${p}_c=0$), with chronic infections (${p}_c>0$) but without any observed mutational burst, and with chronic infections and at least one observed burst. Each measurement was averaged over 5000 simulations. Our results align with clinical data. Namely, the evolutionary rate within a single infected individual was higher than across the population of infected individuals in all cases.

As expected, we found that the evolutionary rate between individuals was highest in populations with chronic infections and where at least one mutational burst was observed. However, even in the absence of a burst, the presence of chronic infections still leads to an increased rate of mutation accumulation compared with populations with only acute infections. Thus, chronic infections could still accelerate pathogen evolution through the generation and transmission of adaptive mutations, even without the production of SARS-CoV-2 VOC-like variants.

Importantly, these patterns are consistent with early pandemic substitution rates under specific parameter regimes. As shown in Supplementary Fig. S11, the dynamics yield accumulated substitutions over 360 days within the empirical range (0.066–0.082 substitutions per genome per day). Dynamics without chronic infections closely match the empirical curve, whereas including chronic infections accelerates evolutionary rates.

## Discussion

In this work, we modelled pathogen evolution within and between hosts including different types of infections: acute, short-term infections, and rare chronic ones. The goal of our study was to understand how chronic infections can influence pathogen evolution over long times. Even with a simple, additive fitness landscape, we found that chronic infections can lead to SARS-CoV-2-like evolutionary dynamics, as mutants with multiple novel mutations arise and spread through the population. Such ‘bursts’ of mutations were especially likely when acute generation times were short.

We found that the frequency of chronic infections had a strong and nonlinear effect on the frequency of mutational bursts. When chronic infections were rare, the number of observed mutational bursts scaled roughly linearly with the frequency of chronic infections. However, frequent chronic infections result in the generation and transmission of more adaptive mutations. Mutants with different beneficial mutations compete for hosts, making it more difficult for a single, dominant variant to quickly emerge.

Our model employs several simplifying assumptions that could be revisited in future work. First, we assumed that the fitness effects of pathogen mutations within and between hosts were the same. While certain mutations, such as those that increase viral load, are highly likely to improve both within-host replication and transmission between individuals, others may only be advantageous in particular scenarios. The distribution of fitness effects of mutations is also challenging to determine. Here, we used data from a recent study of SARS-CoV-2 evolution to parameterize our model (Lee et al. 2025). While the fitness effects of mutations in this study have extensive experimental support, they are subject to noise, and they were determined solely from between-host transmission rather than within-host replication. We have also assumed that the fitness effects of mutations are the same across hosts. Experiments (Doud et al. 2015, Haddox et al. 2018) and computational analyses (Shimagaki et al. 2024) have found many similarities between the fitness effects of mutations for genetically similar viruses, but some differences between hosts would be expected in real scenarios.

Additional features that are currently not included in our model could also promote or discourage the appearance of mutational bursts. Epistasis, i.e. non-additive fitness effects of mutations, was one mechanism originally proposed to explain mutational bursts in SARS-CoV-2 (Zahradník et al. 2021, Ghafari et al. 2022, Martin et al. 2022, Smith and Ashby 2022). Several studies have highlighted epistatic interactions in SARS-CoV-2, especially among Spike mutations in Omicron lineages (Feng et al. 2025, Moulana et al. 2025). Cooperative interactions between mutations could increase the tendency for mutational bursts as viral fitness sharply increases with the addition of multiple mutations. Our model also does not account for feedback between virus transmission and population immunity, which could have complex effects on viral evolution. Post-Omicron, SARS-CoV-2 evolution has appeared more continuous than in the earlier phases of the pandemic (Roemer et al. 2023). However, significantly diverged lineages have also been observed after the emergence of Omicron (Feng et al. 2025), suggesting that mechanisms still exist to generate and spread viral variants that differ significantly from ones that are currently the most dominant in the viral population.

The model that we have developed is a type of ‘metapopulation’ model (Ball et al. 2015), considering evolution both within and between hosts. Past studies have used such models to infer epidemiological dynamics (Stadler et al. 2012) and explain phylogenetic structure (Volz et al. 2012, 2013), among other applications (Frost et al. 2015). Our work contributes to this area by exploring how different types of infections (i.e. acute *versus* chronic infection) contribute to pathogen evolutionary rates.

## Supplementary Material

veag008_supplementary_info

## Data Availability

The data underlying this article are available in the article and in its online supplementary material. Code used in our analysis is available in the GitHub repository at https://github.com/bartonlab/paper-SARS-CoV-2-evolution. The repository also contains the scripts used to process the data and generate the figures in this paper.
