# Supplementary material for: Chronic infections can generate SARS-CoV-2-like bursts of viral evolution without epistasis
Source: Virus Evol. 2026 Feb 18;12(1):veag008. doi: 10.1093/ve/veag008 (PMC13008017; doi:10.1093/ve/veag008)
Supplement: veag008_supplementary_info [file veag008_supplementary_info.pdf]

1. Supplementary Information

Global evolutionary model

Within-host virus evolution model

The viral population, consisting of N infected cells, originates from a single genotype and evolves through discrete and non-overlapping Wright-Fisher generations under selection, mutation, and genetic drift. In each replication cycle, neutral and positive selected mutations are randomly introduced from a binomial distribution with rates $\{\mu_N, \mu_B\}$ respectively. When a mutation occurs in genotype a, its selective effect s_a is sampled from distributions inferred from empirical data (see Mutations fitness landscape subsection). The genotype's fitness is then updated as $f_a = f^{wt} + s_a$, where f^{wt} represents the fitness of the wild-type genotype.

If the population state at generation t is given by the genotype count vector $\vec{x}(t) = (x_1(t), \ldots, x_M(t))$, where $x_a(t)$ denotes the number of cells with genotype a, selected species in the next generation are given by multinomial sampling:

$$P(\vec{x}(t+1)|\vec{x}(t)) = N! \prod_{a=1}^{M} \frac{p_a^{x_a(t+1)}}{(x_a(t+1))!}, \quad \textbf{(S1)}$$

where the success probability of each genotype is given by its relative fitness, $p_a = f_a / \sum_b f_b$.

Between-host virus evolution model

We model the spread of the virus as a branching process where each infected individual can generate n additional cases. To account for superspreading—where a small fraction of infectious hosts are responsible for most transmissions—the number of secondary infections per infected individual is drawn from a negative binomial distribution:

$$P_{NB}(n|k, R^i) = \frac{\Gamma(k+n)}{n!\Gamma(k)} \left(\frac{k}{k+R^i}\right)^k \left(\frac{R^i}{k+R^i}\right)^n \quad \textbf{(S2)}$$

where k is the dispersion parameter, and $R^i = \bar{R}\left(1 + \langle f \rangle^i\right)$ represents the effective reproductive number associated with host i. This quantity depends on the average reproductive number ($\bar{R}$) and the average fitness of the donor host virus population, $\langle f \rangle^i = \sum_{a=1}^{N} p_a f_a^i$, where p_a is the frequency of the variant. After transmission, donors are assumed to be recovered and removed from the current infected population. Acute and chronic cases are indistinguishable within their respective groups, as cross-immunity is not considered. This ensures that transmission dynamics remains the primary focus of our approach. New infections are established by a single virion sampled from the donor quasispecies distribution, and most of the variant diversity previously generated is lost. This mimics the characteristic narrow transmission bottleneck observed in respiratory viruses such as SARS-CoV-2. Variants with higher fitness can persist through the transmission bottleneck only if a sufficient intrahost evolution time allows their frequency to rise. However, the most common scenario (with acute infections) is that variants without strong selective advantage overcome the transmission bottleneck by chance, a phenomenon of genetic drift.

Mutational fitness landscape

The distribution of neutral and beneficial mutation effects during within-host replication was derived from selection coefficients inferred from SARS-CoV-2 temporal genomic data (Lee et al. 2025), as shown in **Supplementary Fig. S1**. In this study, an additive fitness landscape was reconstructed using an epidemiological model of viral transmission, similar to the one described by Eq. (S2). The method involves inverting the relationship between mutation histories and the model's selection parameters, which quantify the effects of mutations on viral transmission.

In our model, the distribution of neutral mutations is obtained by fitting a normal distribution to the values of the selection coefficients in the range -0.02 to 0.02. This choice is grounded in population genetics, where, under weak selection, mutation effects are expected to be symmetrically distributed around zero due to stochastic processes such as genetic drift. For beneficial mutations, we fit a log-normal distribution to values greater than 0.02, as these mutations are expected to follow a heavy-tailed distribution: most have small positive effects, while some confer substantial fitness advantages. The log-normal distribution naturally captures this asymmetry, ensuring that inferred beneficial effects remain positive and preventing biologically unrealistic negative values. Here we emphasize that our main goal is to employ distributions of mutational effects in our simulations that are qualitatively consistent with measurements from biological data, rather than rigorously characterizing the distribution of inferred selection coefficients.

Dynamic simulations

We implemented the global evolutionary model in Julia. We ran multiple independent realizations of the evolutionary model over 1000 days, for the intra-host viral population size $N = 1000$ (Bar-On et al. 2020). This value ensures that genetic drift does not overwhelmingly dominate selection, allowing beneficial mutations to increase in frequency while still accounting for

stochastic effects. To model generation times for chronic cases, we assume a log-normal distribution with mean $\mu_L = 150$ days and standard deviation $\sigma_L = 80$ days. This choice reflects the pronounced right skew suggested by reported durations of chronic SARS-CoV-2 infections (Choi et al. 2020; Avanzato et al. 2020; Cele et al. 2022; Chaguza et al. 2023), yielding a median of ~ 132 days and a 95th percentile of ~ 301 days, thereby encompassing both typical multi-month infections and rare extended cases. Sensitivity analyses (see **Supplementary Fig. S6** and **Supplementary Fig. S7**) confirmed the robustness of our main results across alternative distributions and parameter ranges. The dispersion parameter in the negative binomial distribution was set to $k = 1.0$, which is within the estimated range for SARS-CoV-2 (Wegehaupt et al. 2023). We choose this moderate value to reflect that, in our model, transmission heterogeneity is not driven solely by k, as we consider an effective reproductive number R^i dependent on viral fitness in the donor, which also contributes to the non-homogeneous spread of secondary infections.

In the between-host model, the population size of infected individuals is not fixed. If the population is too small, stochastic extinction becomes likely; if it is too large, sampling from the negative binomial distribution becomes computationally demanding. To balance these considerations, we introduce a population size threshold M, such that the population is allowed to grow until it reaches M, at which point the reproduction number R^i is dynamically adjusted to keep the average reproduction number close to 1. This results in an asymptotic population size that remains approximately stable over time. Throughout the main text, we present results for $M = 300$, which enables efficient exploration of the parameter space. However, we also include results for a more realistic population size of $M = 10^4$ in **Supplementary Fig. S8**, demonstrating that the qualitative behavior of the model is preserved.

Burst detection method

To identify bursts of mutations along the trajectories of accumulated mutations, we follow a step-by-step process. Initially, we calculate the slope at each time point for M trajectories obtained from simulations involving only acute cases. Subsequently, we apply a Savitzky–Golay filter (Savitzky and Golay 1964) with a time window length (w) and polynomial order (p) to smooth the slope time series for each simulation. We extract the maximum values from the smoothed slope time series and use them to build a Gaussian null distribution (see **Supplementary Fig. S12a**).

We use the same smoothing process for the slope time series of simulations involving chronic disease cases. We then calculate the z-score for each time point using the mean and standard deviation obtained from the previously established null distribution. Mutation bursts are identified as outliers in this null distribution, defined by instances where the z-score exceeds 3.5. Given that multiple time points near the jump meet this criterion, we identify change points in the z-score time series. These change points delineate the start and end times of each jump, with the midpoint representing the burst time. The entire procedure is summarized in **Supplementary Fig. S12b**.

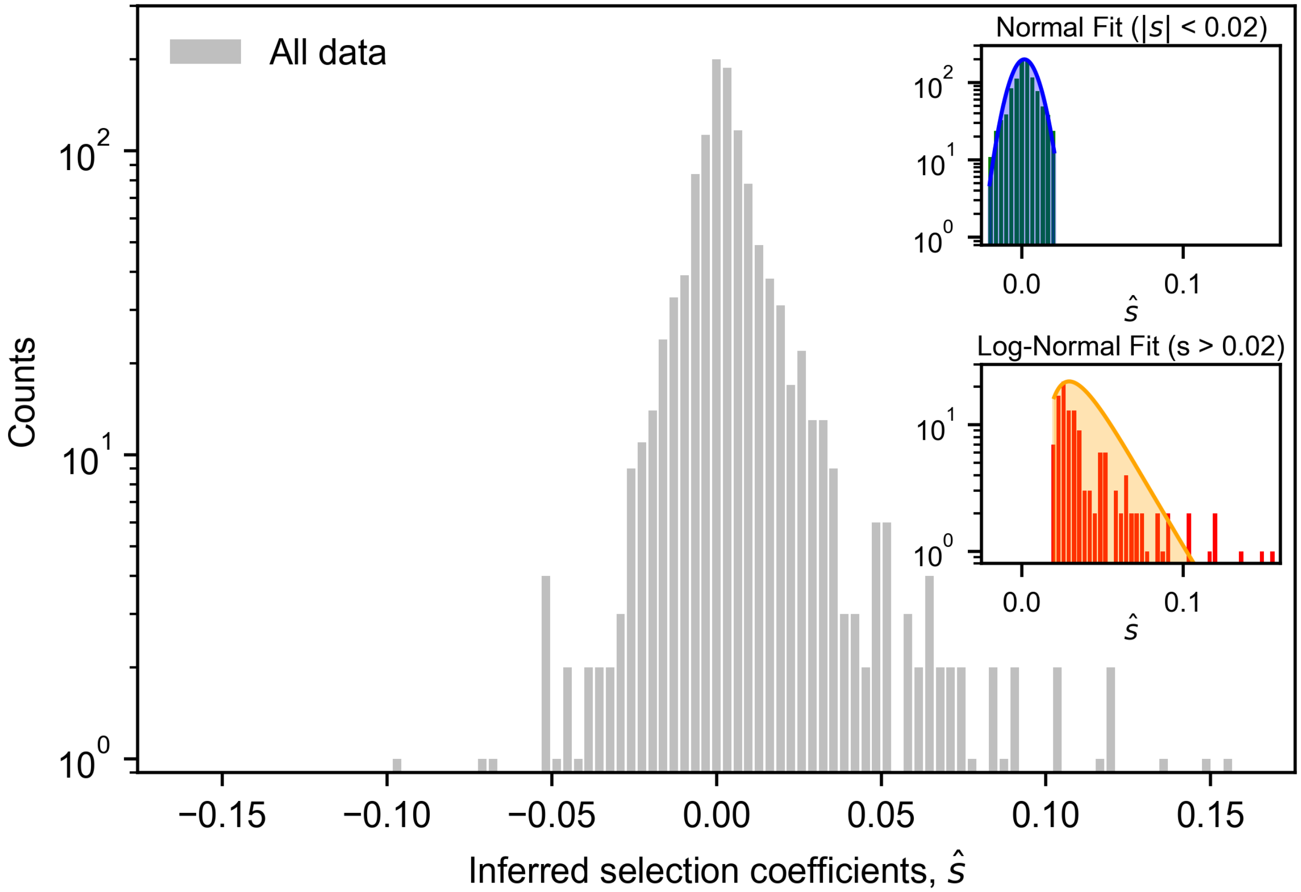

Fig. S1. Inferred transmission effects of SARS-CoV-2 mutations. The main plot displays a histogram of selection coefficient values inferred from SARS-CoV-2 temporal genomic data (Lee et al. 2025). The top-right inset plot shows the normal distribution fit for coefficient values considered neutral $(-0.02 < \hat{s} < 0.02)$; from this distribution, neutral mutation effects during within-host evolution were sampled. The bottom-right inset plot shows the log-normal distribution fit for values greater than 0.02, representing significantly beneficial mutations; from this distribution, beneficial mutation effects during within-host evolution were sampled.

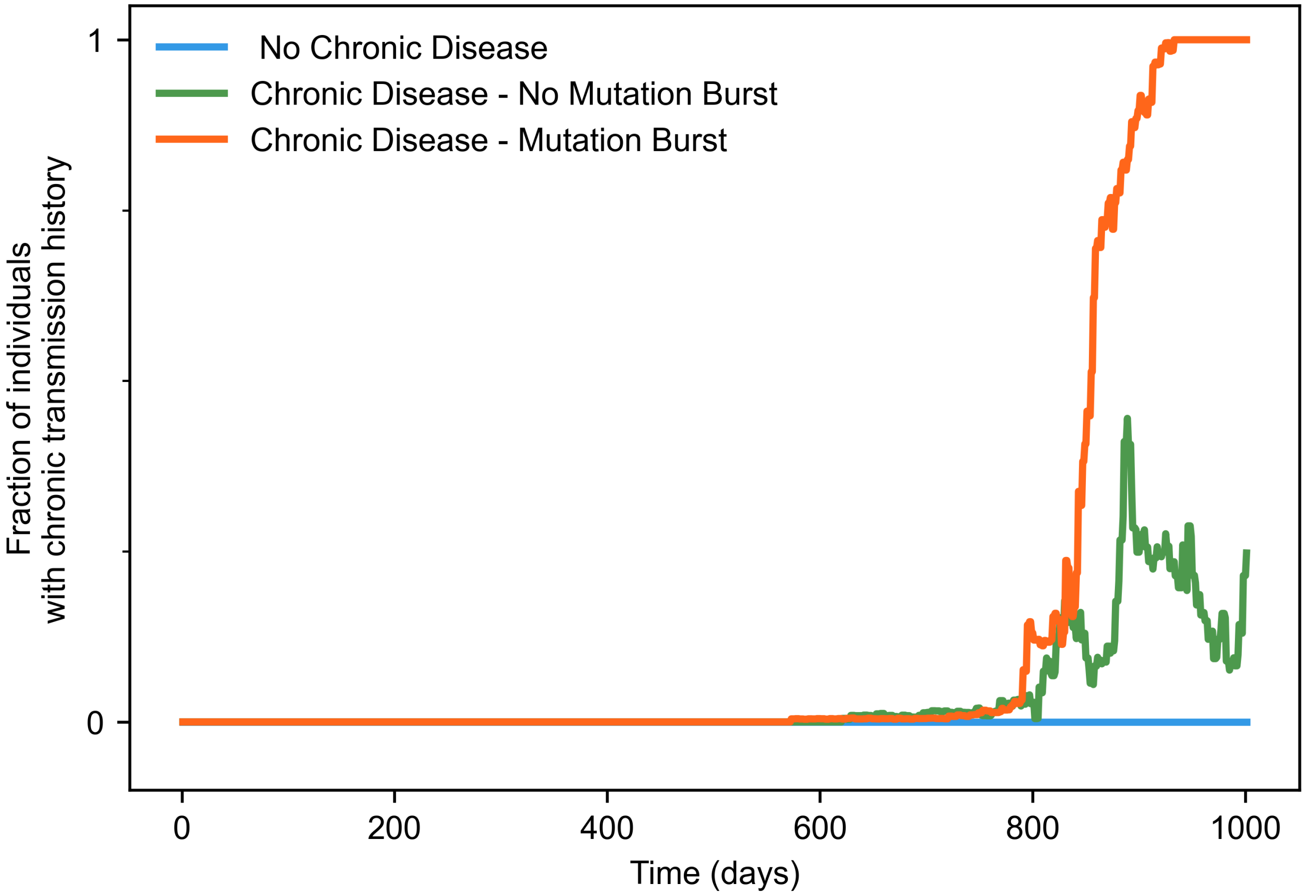

Fig. S2. Fraction of individuals with variants transmitted through chronic infection. Each curve represents the fraction of individuals infected by variants that have passed through individuals with chronic infections, based on the dynamics shown in **Fig. 3**.

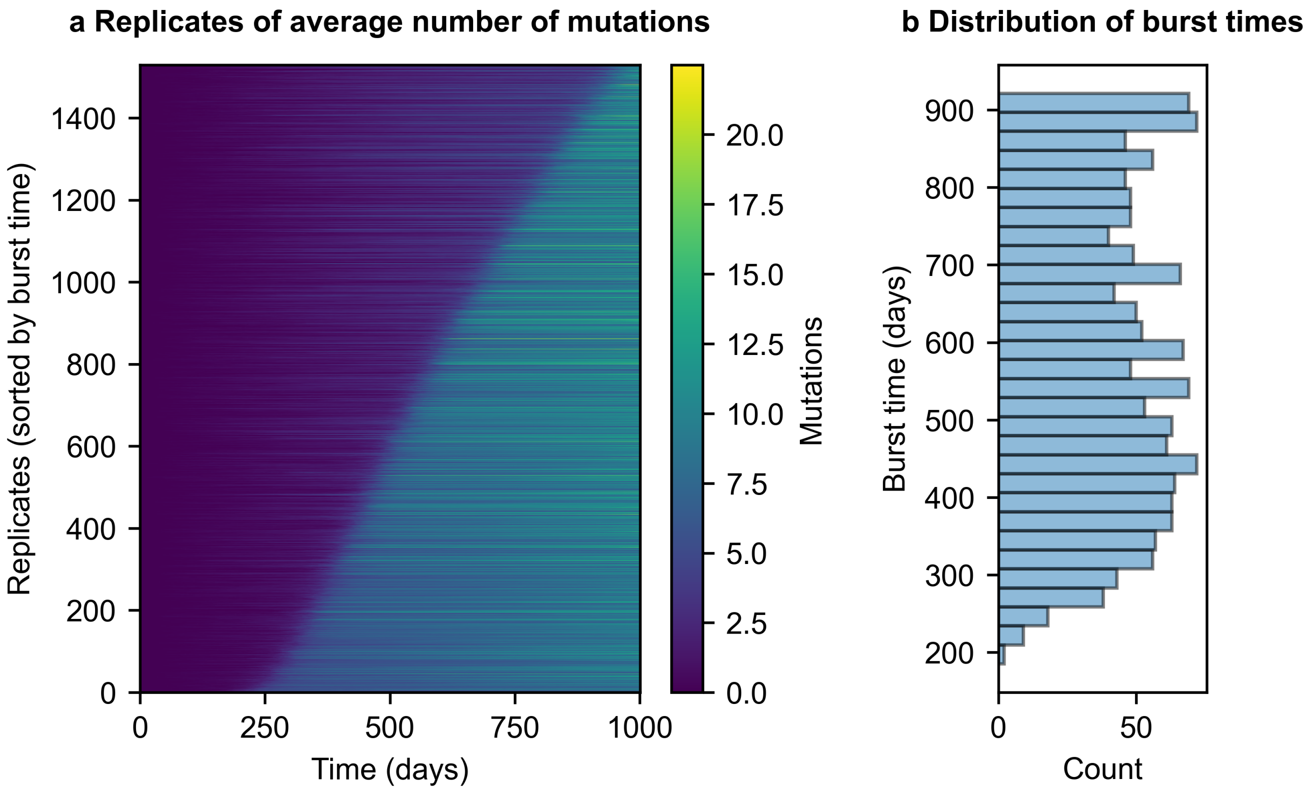

Fig. S3. Burst-time variability across replicate simulations. a, Heatmap of the average number of accumulated mutations for 1000 replicate simulations, with rows sorted by each replicate's burst time. **b**, Corresponding histogram summarizing the burst-time distribution. Simulation parameters match those in **Fig. 3.c**

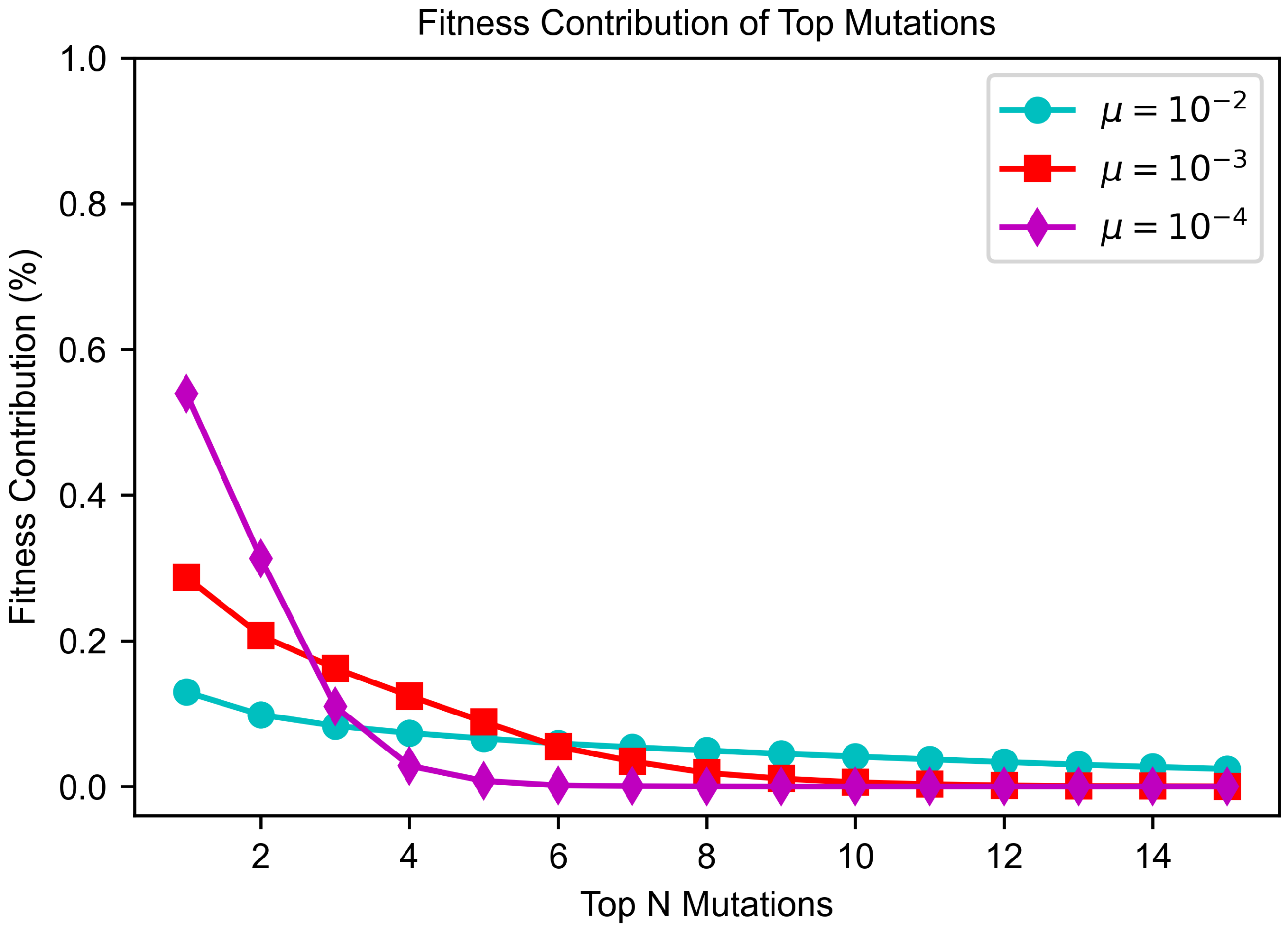

Fig. S4. Contribution of the top mutations to the fitness of variants that dominate the population after the burst. Each curve represents the average over 1,000 simulations for beneficial mutation rates $\mu_B = \{10^{-2}, 10^{-3}, 10^{-4}\}$ mutations/cycle and a neutral mutation rate of $\mu_N = 10^{-4}$ mutations/cycle. Chronic cases are included at a percentage per transmission event of $p_c = 10^{-4}$, with generation times t_c drawn from a log-normal distribution with mean $\mu_L = 150$ days and standard deviation $\sigma_L = 80$ days. In all cases, the generation time for acute infections is fixed at $t_a = 2$ days.

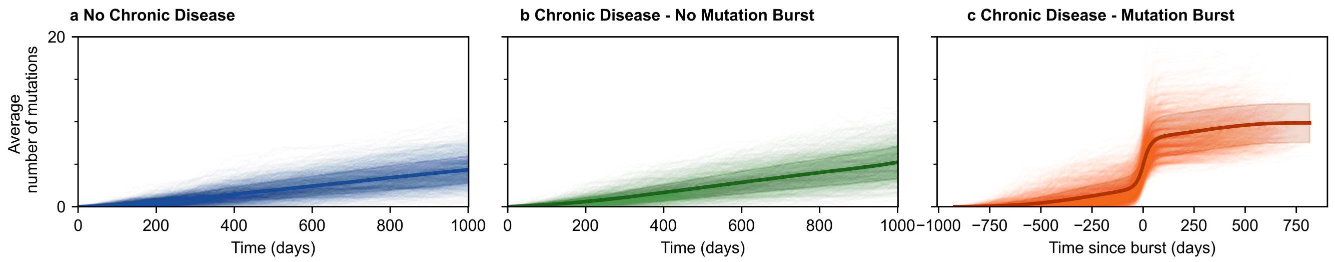

Fig. S5. Variability in mutation accumulation across replicate simulations. Each panel shows 1000 replicate simulations corresponding to the scenarios in Fig. 3: **a**, acute-only infections, **b**, mixed acute–chronic infections without a burst, and **c**, mixed acute–chronic infections with a burst. Curves show the average number of accumulated mutations in each replicate, with lightly colored lines indicating individual trajectories and a mean ± standard deviation (SD) ribbon summarizing variability. In panel **c**, burst timing varies across replicates; to better visualize differences in burst magnitude, trajectories are aligned by each replicate's burst time. Simulation parameters match those in **Fig. 3**.

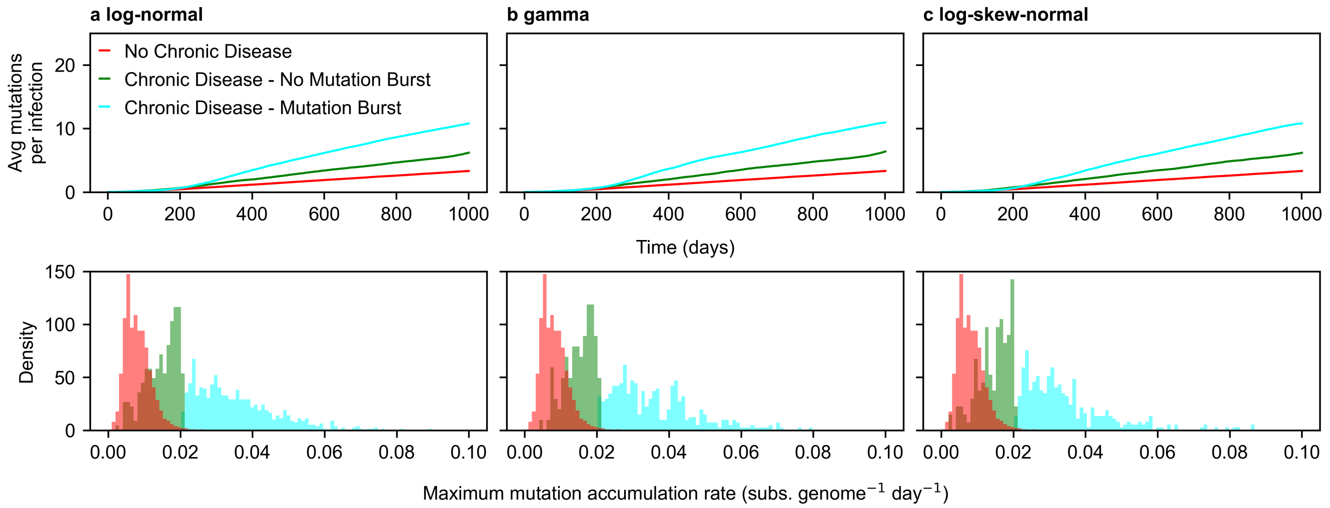

Fig. S6. Sensitivity analysis of between-host evolutionary rates under alternative chronic-infection generation time distributions. Each column corresponds to a different distribution for chronic-infection generation times—**a**,log-normal, **b** gamma and **c**, log-skew-normal—all with the same mean (150 days) and standard deviation (80 days). The top row shows the average number of accumulated mutations per infection over 1000 simulations, comparing between-host evolution with and without chronic infections, analogous to **Fig. 5**. The bottom row displays the distribution of maximum slopes for the corresponding accumulated mutation curves. These results indicate that the main patterns of mutation accumulation and between-host evolutionary dynamics are consistent across distributions.

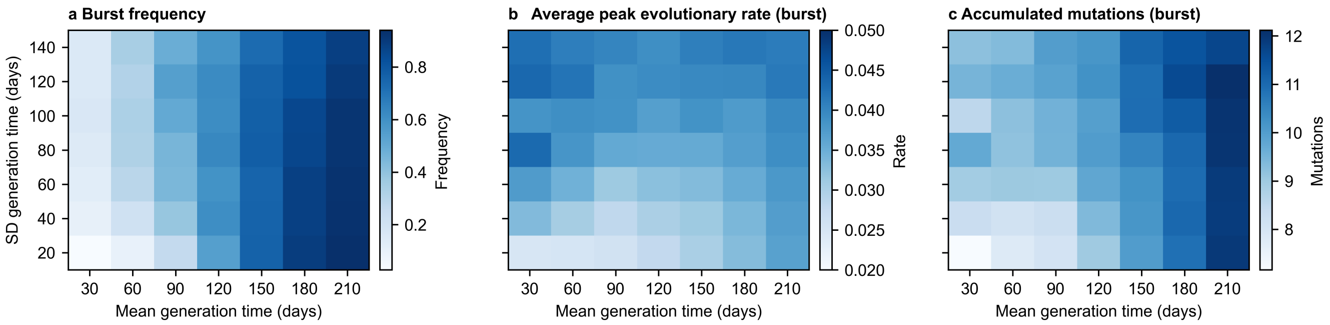

Fig. S7. Sensitivity analysis of evolutionary dynamics under variation in chronic-infection generation time parameters. Chronic-infection generation times follow a log-normal distribution, and we vary their mean and standard deviation. **a**, Fraction of simulations exhibiting at least one burst event. **b**, Average peak mutation-accumulation rates for burst trajectories. **c**, Final number of accumulated mutations for dynamics that include at least one burst event.

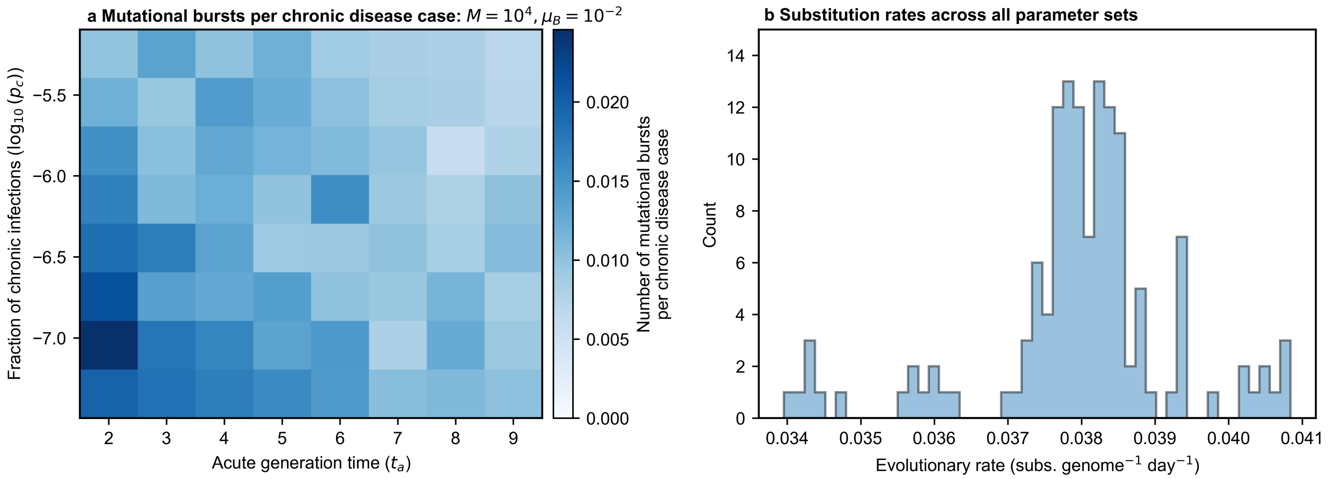

Fig. S8. Number of mutational bursts per chronic disease case and substitution rates across parameter sets for beneficial mutation rate of 10^{-2} mutations/cycle and individual population size of $M = 10^4$. **a**, Heatmap showing the mean number of mutational bursts per chronic disease case across 64 parameter combinations of acute generation time and the fraction of individuals with chronic infections. Each value represents an average over 5000 simulations. **b**, Distribution of average substitution rates computed for the same parameter sets shown in panel (a), including dynamics without chronic disease, with chronic disease but without mutation bursts, and with chronic disease and mutation bursts.

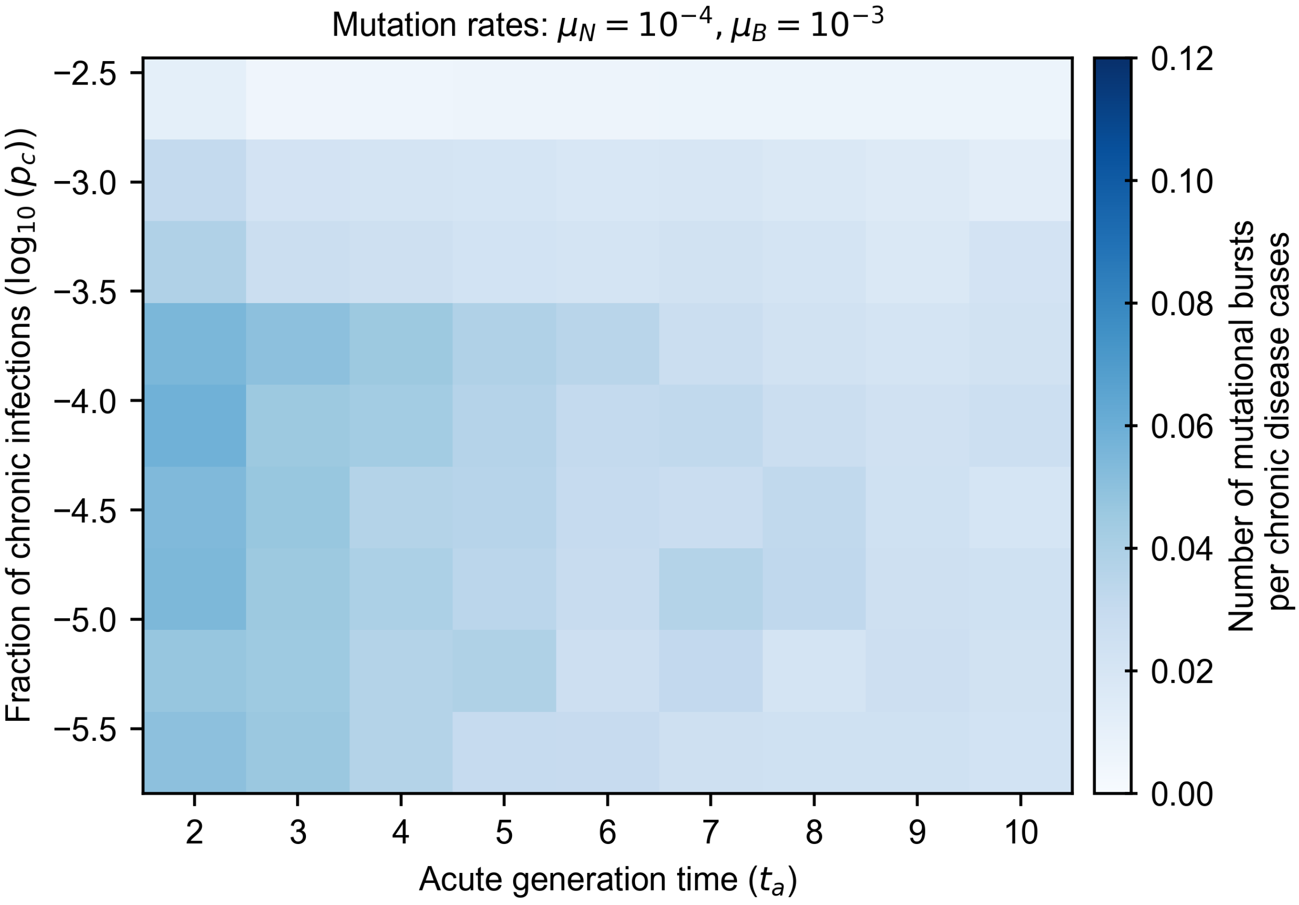

Fig. S9. Number of mutational bursts per chronic disease case for beneficial mutation rate of 10^{-3} mutations/cycle. This figure is analogous to **Fig. 4** in the main text, but with a lower beneficial mutation rate.

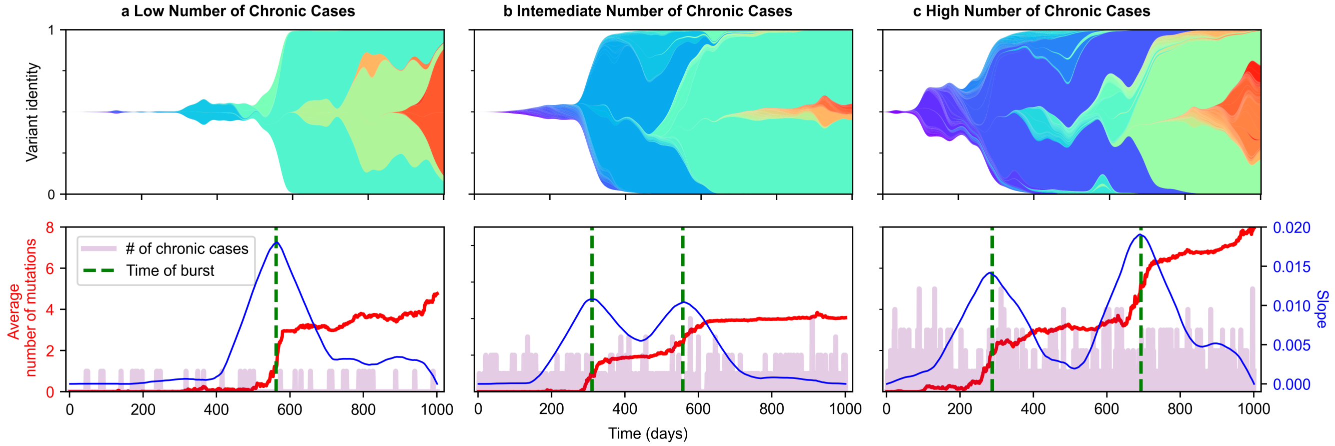

Fig. S10. Dynamic evolution of the viral population under varying chronic infection probabilities. a, Low number of chronic cases, corresponding to a probability per transmission event of $p_c = 4 \times 10^{-4}$. **b**, Intermediate number of chronic cases, with a probability per transmission event of $p_c = 3.7 \times 10^{-3}$. **c**, High number of chronic cases, resulting from a probability per transmission event of $p_c = 7.0 \times 10^{-3}$. For all simulations, we consider beneficial and neutral mutation rates $\mu_B = 10^{-4}$ mutations/cycle and $\mu_N = 10^{-4}$ mutations/cycle, respectively. Generation times are set at $t_a = 2$ for acute cases, while for chronic cases, they follow a log-normal distribution with a mean of $\mu_L = 150$ days and a standard deviation of $\sigma_L = 80$ days.

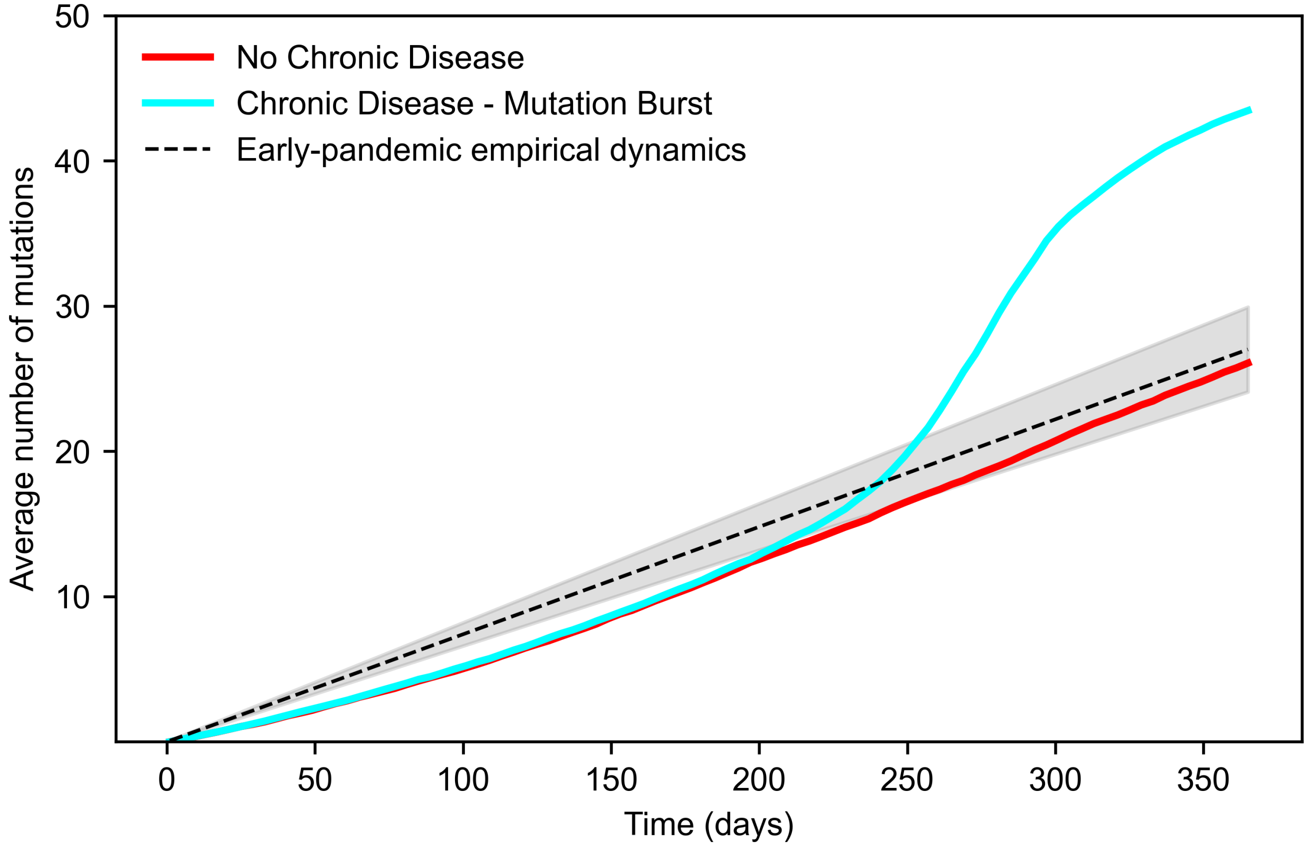

Fig. S11. Early-pandemic evolutionary dynamics reproduced by the model. Accumulated number of substitutions over time for dynamics without chronic infection (red) and with chronic infection and mutation bursts (cyan), assuming $M = 10^4$ and beneficial mutation supply rate of $\mu_B = 0.025$. Dashed lines indicate the empirical early-pandemic substitution rate range (0.066–0.082 substitutions per genome per day), shown as the expected accumulated substitutions over time, with the shaded region representing uncertainty. Under these conditions, the model reproduces early-pandemic evolutionary dynamics over the first year of circulation, while showing that chronic infections accelerate evolutionary rates relative to dynamics without chronic infection.

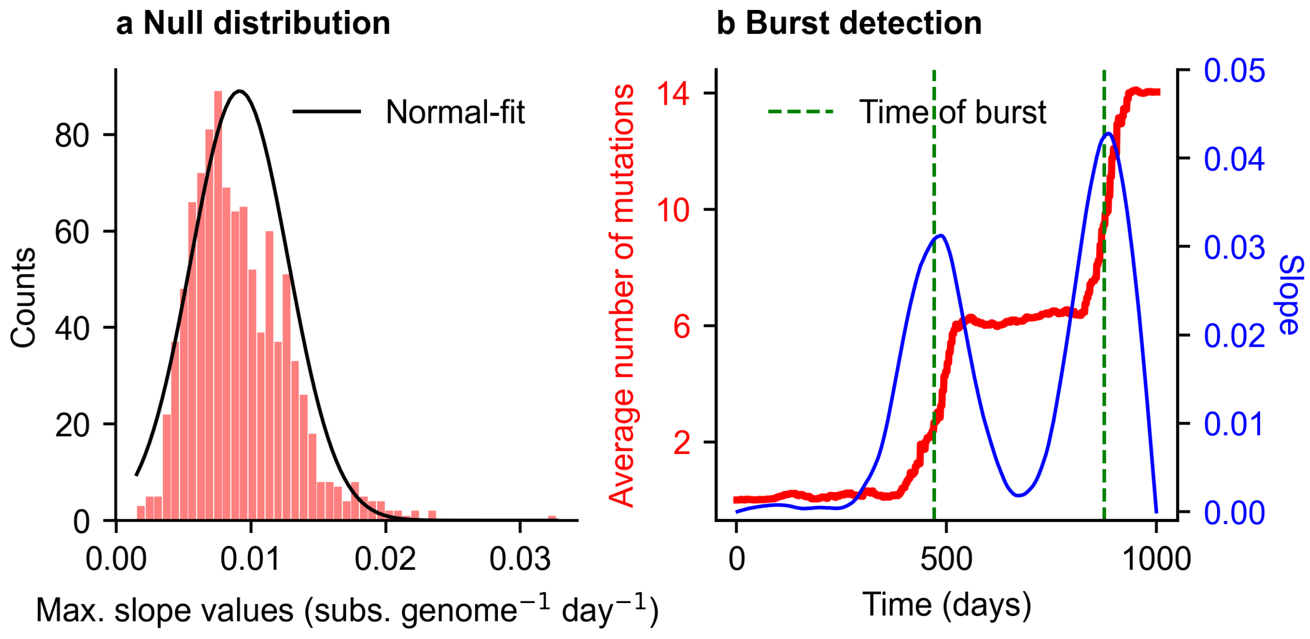

Fig. S12. Detection of mutation bursts. a, Distribution of maximum slopes of accumulated mutation trajectories without chronic infection for acute generation time of $t_a = 4.0$ days and mutation rates: $\mu_B = 10^{-3}$ beneficial mutations per cycle and $\mu_N = 10^{-4}$ neutral mutations per cycle. **b,** For a simulation with chronic infection fraction $p_c = 10^{-4}$, the number of accumulated mutations averaged over an individual's population is shown in red. The blue curve indicates the smoothed slope time series with two peaks, detected by z-score time series change points and represented by the vertical green dashed lines. For smoothing using the Savitzky–Golay filter, we use parameters $w = 150$ and $p = 1$.

Supplementary References

Avanzato VA, Matson MJ, Seifert SN, Pryce R, Williamson BN, Anzick SL, et al. Case Study: Prolonged Infectious SARS-CoV-2 Shedding from an Asymptomatic Immunocompromised Individual with Cancer. Cell 2020;183(7):1901–1912.e9. https://www.sciencedirect.com/science/article/pii/S0092867420314562.

Bar-On YM, Flamholz A, Phillips R, Milo R. Science Forum: SARS-CoV-2 (COVID-19) by the numbers. eLife 2020 mar;9:e57309. https://doi.org/10.7554/eLife.57309.

Cele S, Karim F, Lustig G, San JE, Hermanus T, Tegally H, et al. SARS-CoV-2 prolonged infection during advanced HIV disease evolves extensive immune escape. Cell Host & Microbe 2022;30(2):154–162.e5. https://doi.org/10.1016/j.chom.2022.01.005.

Chaguza C, Hahn AM, Petrone ME, Zhou S, Ferguson D, Breban MI, et al. Accelerated SARS-CoV-2 intrahost evolution leading to distinct genotypes during chronic infection. Cell Rep Med 2023 Feb;4(2):100943.

Choi B, Choudhary MC, Regan J, Sparks JA, Padera RF, Qiu X, et al. Persistence and Evolution of SARS-CoV-2 in an Immunocompromised Host. New England Journal of Medicine 2020;383(23):2291–2293. https://doi.org/10.1056/NEJMc2031364, pMID: 33176080.

Lee B, Sohail MS, Finney E, Ahmed SF, Quadeer AA, McKay MR, et al. Inferring effects of mutations on SARS-CoV-2 transmission from genomic surveillance data. Nature Communications 2025;16(1). https://doi.org/10.1038/s41467-024-55593-0.

Savitzky A, Golay MJE. Smoothing and Differentiation of Data by Simplified Least Squares Procedures. Analytical Chemistry 1964 Jul;36(8):1627–1639. https://doi.org/10.1021/ac60214a047.

Wegehaupt O, Endo A, Vassall A. Superspreading, overdispersion and their implications in the SARS-Cov-2 (COVID-19) pandemic: a systematic review and meta-analysis of the literature. BMC Public Health 2023;23(1):1003. https://doi.org/10.1186/s12889-023-15915-1.
